# Short Term Minimum Water Temperatures Determine Levels of Infection by the Amphibian Chytrid Fungus in *Alytes obstetricans* Tadpoles

**DOI:** 10.1371/journal.pone.0120237

**Published:** 2015-03-20

**Authors:** Saioa Fernández-Beaskoetxea, Luis M. Carrascal, Andrés Fernández-Loras, Matthew C. Fisher, Jaime Bosch

**Affiliations:** 1 Museo Nacional de Ciencias Naturales, CSIC, Madrid, Spain; 2 Department of Infectious Disease Epidemiology, Imperial College London, St. Mary’s Hospital, London, United Kingdom; 3 Centro de Investigación, Seguimiento y Evaluación, Parque Nacional de la Sierra de Guadarrama, Rascafría, Spain; University of Regina, CANADA

## Abstract

Amphibians are one of the groups of wildlife most seriously threatened by emerging infectious disease. In particular, chytridiomycosis, caused by the aquatic fungus *Batrachochytrium dendrobatidis*, is responsible for amphibian species declines on a worldwide scale. Population-level outcomes following the introduction of the pathogen are context dependent and mediated by a large suite of abiotic and biotic variables. In particular, studies have shown that temperature has a key role in determining infection dynamics owing to the ectothermic nature of the amphibian host and temperature-dependency of pathogen growth rates. To assess the temperature-dependent seasonality of infectious burdens in a susceptible host species, we monitored lowland populations of larval midwife toads, *Alytes obstetricians*, in Central Spain throughout the year. We found that infections were highly seasonal, and inversely correlated against water temperature, with the highest burdens of infection seen during the colder months. Short-term impacts of water-temperature were found, with the minimum temperatures occurring before sampling being more highly predictive of infectious burdens than were longer-term spans of temperature. Our results will be useful for selecting the optimal time for disease surveys and, more broadly, for determining the key periods to undertake disease mitigation.

## Introduction

Spatiotemporal variation in the amount of clinical disease caused by pathogens is common in nature [1–4]. In many settings, this variation follows a marked seasonal pattern that is related to the thermal requirements of the pathogens [5–6] or to changes in the host density and its immune system [7–10]. Seasonality determines much of the external environmental variation influencing diseases, and several studies have shown that seasonality plays an important role in population ecology of both host and pathogen [11–12]. Therefore, by studying seasonal environmental variability it is possible to make predictions about changes in infection dynamics over time.

Chytridiomycosis is an amphibian specific disease that has been recognized as one of the main causes of recent amphibian declines and extinctions worldwide [13–14]. The causal agent, *Batrachochytrium dendrobatidis* (*Bd*) is a pathogenic fungus whose impacts on amphibian populations show strong spatial and temporal variation. Spatial variation in the prevalence of infection is highly heterogeneous and caused by taxonomic (under- and over-infected families), environmental (e.g., temperature range and precipitation at a site), and community-level (e.g., species richness) determinants [14]. It is well known that some populations of susceptible species do not develop any symptoms of disease, even when many individuals are highly parasitised by *Bd* [15], whereas others suffer severe declines or extinctions. This variation in the virulence of *Bd* is known to be caused by a complex interaction between biotic (host community structure, predatory microbiota, probiotic skin bacteria) and abiotic (altitude, temperature, UVB, climatic seasonality) factors [16–21]. In temperate zones, with greater seasonality, the outcome of infection is usually more variable than in tropical areas [22], being more harmful in cool and high altitude regions [23–25]. Within a region, seasonal environmental fluctuations have important consequences for the development of disease.

Among the many environmental factors that regulate populations of saprobic aquatic fungi, temperature has profound impacts on their population dynamics and explains why many chytridiomycetes bloom with seasonal temperature changes [26]. *Bd in vitro* grows most rapidly between 17°C and 25°C. At temperatures above 28°C and below 10°C the growth rate decreases [27–28] and the pathogen is killed within short periods of time at temperatures of 37°C or above [29]. An increasing number of studies have revealed a strong influence of temperature on patterns of *Bd* infection in wild populations [18, 30–32]. Australian adult frogs show peaks of prevalence in the coolest months of the year [18, 33–35] and the same pattern is known to occur in North America and Puerto Rico, where the epidemiology of *Bd* is strongly dictated by seasonality, transitioning from complete disappearance during summer to causing die-offs during winter [22, 36–40].

While *Bd* attacks the keratinized mouthparts of amphibian larvae, tadpoles survive as reservoirs without suffering obvious symptoms of disease [16, 23]. However, when metamorphosis occurs, the fungus invades the recently keratinized skin of the juvenile animal causing hyperkeratosis, impairment of osmoregulatory processes and triggering death in vulnerable species [43]. Mortality is dependent on an individuals intensity of infection, which also shows temporal variation depending on the year, breeding behaviour of the animals, density of the host or their body size and sex [41–42]. Therefore, linking changing patterns of infection intensity to biotic and abiotic factors is important for understanding the rate of clinical disease that ultimately impacts host populations. In this study we aimed to assess the temperature-dependent seasonality of infectious burdens in tadpoles of a susceptible species in order to improve our ability to model and predict the impacts of chytridiomycosis across host populations.

## Materials and Methods

The study area is located in the agropastoral valley of the Duoro River in the municipality of Toro (Province of Zamora in Castilla-León, Spain). Fresh water springs are common and many flow into artificial troughs that are used by cattle. *Aytes obstetricans* (the common midwife toad) breeds in these troughs, where the species reproduces twice a year (spring and autumn) and autumn tadpoles often overwinter.

Six troughs located between 693 and 772 m of altitude were selected for the study (Nueva de Bardales, UTM coordinates 30T 304137, 4588429; Valdespino, UTM coordinates 30T 300479, 4587707; Villares, UTM coordinates 30T 299974, 4586932; Perros, UTM coordinates 30T 297429, 4585493; Picarico, UTM coordinates 30T 295107, 4586056 and Marlota, UTM coordinates 30T 294955, 4583532). Selected troughs were similar in size and hold similar tadpole densities to those found across the Iberian Peninsula. Submerged dataloggers (HOBO Pro v2 Water Temperature Logger U22–001, Onset Inc., Bourne, Massachusetts) in each trough provided a continuous half-hourly measurement of water temperature across one year, from February of 2010 to January of 2011. These sites were not privately owned or protected and therefore no permissions to access the troughs are required. On the other hand, because the studied species is protected by national legislation, animal care as well field permits to conduct this research were obtained from the Consejería de Medio Ambiente, Junta de Castilla y León, Spain (permit EP/CyL/20/2010).

Each month, we estimated the total number of *A*. *obstetricans* tadpoles present in every trough, as well the proportion of tadpoles below Gosner stage 36 (no, or rudimentary, hind limbs present)[44]. Twenty tadpoles were captured at random with a small hand-net and swabbed gently with a sterile cotton swab (MW 100–100, Medical Wire & Equipment) 20 times across their mouthparts. After swabbing, the tadpoles were released back into their troughs. According to the current national legislation no approval from an Animal Care and Use Committee is required for these procedures. DNA extractions from the swabs were performed using PrepMan Ultra (Applied biosystems) and the amount of *Bd* DNA present in each sample was measured through a CFX96TM Real-Time PCR Detection System (BIO-RAD) with a *Bd*-specific Taqman Assay [45]. Each 96-well assay plate included a negative control and four different standards containing DNA from 100, 10, 1 and 0.1 *Bd* genome equivalents. We used an isolate from infected *A*. *obstetricans* in Northwest Spain (IA042, Ibón Acherito, Spanish Pyrennees) as a source of standards. Both, the standard isolate and the strain present at the studied area genetically closely related, being members of the *Bd*GPL lineage (unpublished results).

Infection load was measured as the number of zoospore equivalents per swab. Each sample was performed in duplicate and individuals were considered *Bd-*positive when the results of the two replicates were consistent and > 0.1 zoospore genome equivalents. If not, the sample was re-run and considered positive only if another positive result occurred. Prevalence was calculated as the percentage of infected individuals.

General Linear Mixed Models were applied to analyse variation in population averaged *Bd* loads (log transformed; x’ = ln[x+1]) throughout eleven months (from March to January). Population averaged *Bd* loads were used instead of individual values as our sampling unit was the population at each “site”. Site was considered as a random factor, and average minimum and maximum daily water temperatures, tadpole abundance and tadpole development as covariates. These analyses were carried out using estimates of average minimum and maximum temperatures across six different periods of time (2, 5, 10, 15, 22 and 30 days prior to tadpole sampling). As February was the first month with temperature records we could not include this month in data analyses due to the lack of temperature data for January 2010. The mean square (MS) and the degrees of freedom (df) of the error terms were estimated following Satterthwaite’s method; this finds the linear combinations of sources of random variation that serve as appropriate error terms for testing the significance of the respective effect of interest. We chose this design, instead of random intercept + random slope models due to (a) the lack of significance of interaction terms with the random factor and (b) the analytical power. As the interaction terms [site x minimum temperature], [site x maximum temperature], [site x tadpole abundance] and [site x tadpoles development] were not significant, there is no need to develop a random intercept + random slope model. Further, random intercept + random slope models demand large sample sizes in order to attain high levels of statistical power; this is not our case, as we only have six different sites sampled in eleven months.

Standardized regression coefficients (β) were obtained for covariates as a measure of the sign and magnitude effects of predictor variables (i.e. analyses were carried out with standardized variables, such that their averages are zero and variances are one). For the random factor “site” we estimated the proportion of variance accounted for in each model. Homoscedasticity and normality of residuals of the General Linear Mixed Models were checked and did not deviate from the canonical assumptions.

Seven alternative models were compared with Akaike’s second-order AIC corrected for small sample sizes (AICc) [46] to assess their weights of evidence. All these models included the site, tadpole abundance and tadpole development as predictors, but varied according to the inclusion of temperature covariates. Six *a priori* models included the average minimum and maximum temperatures estimated across six different periods of time; 2, 5, 10, 15, 22 and 30 days before tadpole sampling. The seventh model did not include any temperature measurement, to control against the influence of temperature in determining the population averaged *Bd* loads of tadpoles (see Table 1). The strength of evidence of models was obtained using weights (Wi) derived from AICc figures, and their quotients were used to compare pairs of models. Parameter estimates (β, proportion of variance accounted for by “site” and R2) were averaged using model weights (Wi). Such a model-averaged estimator compares favorably in terms of bias and precision against a single estimator extracted from the single best model. Finally, all possible subsets of the predictors using General Linear Mixed Models were estimated, considering each time span (2, 5, 10, 15, 22 and 30 days prior to tadpole sampling) and always including the random factor “site” (76 models; to control for the fact that our true sample unit was the population at each “site”).

**Table 1:** Alternative models for *Batrachochytrium dendrobatidis* infection loads (in logarithm) in six different sites.

	AICc	W_i_	W_t_ / W	model R^2^	Min T	Max T	abund	develop	%var sites
with temperature-2 days	181.82	0.444	264284	0.523	-0.780	0.082	-0.013	-0.037	0.053
with temperature-5 days	182.08	0.389	231832	0.522	-0.622	-0.079	-0.009	-0.039	0.052
with temperature-10 days	184.25	0.131	78112	0.506	-0.392	-0.289	-0.024	-0.029	0.050
with temperature-15 days	187.54	0.025	15085	0.480	-0.281	-0.378	-0.024	-0.024	0.050
with temperature-22 days	189.85	0.008	4766	0.462	-0.106	-0.529	-0.029	-0.006	0.049
with temperature-30 days	192.50	0.002	1268	0.440	-0.067	-0.546	-0.024	0.020	0.048
without temperature	206.79	0.000		0.242			-0.451	0.087	0.038
weighted averages				0.519	-0.648	-0.047	-0.013	-0.036	0.052

The first six models include the average minimum and maximum water temperature (T) in six different time spans prior to tadpole sampling (i.e., two, five, ten, 15, 22 and 30 days), taking into account other three predictor terms: tadpole abundance (abund), tadpole development (develop) and site. Sample size is 66 (six sites x 11 months). AICc: AIC corrected for small sample sizes. W_i_: model weights. W_t_ / W: quotient of strength of evidence dividing the weight (W_t_) each model containing both maximum and minimum temperatures with the model without temperatures (W). Figures below Min T, Max T, tadpole abundance and development are standardized regression coefficients (β) obtained in mixed general linear models (β values inform about the magnitude and sign of the partial relationships of the predictor variables). Weighted averages: multimodel inference of standardized β regression coefficients considering the model weights W_i_. The AICc figure for the null model (i.e., not including any effect) is 208.04.

The influence of phenology on the population averaged *Bd* loads in tadpoles was tested by means of a three-order polynomial for month (March-1, January-11) using the residuals of the seven *a priori* models in Table 1. These post-hoc models attempt to quantify the magnitude of variance in population averaged *Bd* loads that occur across the year, but are not related to temporal changes in temperature, tadpole abundance and tadpole development.

All the statistical analyses were carried out using STATISTICA 10 (StatSoft Inc, Tulsa, Oklahoma).

## Results

**Fig 1:**
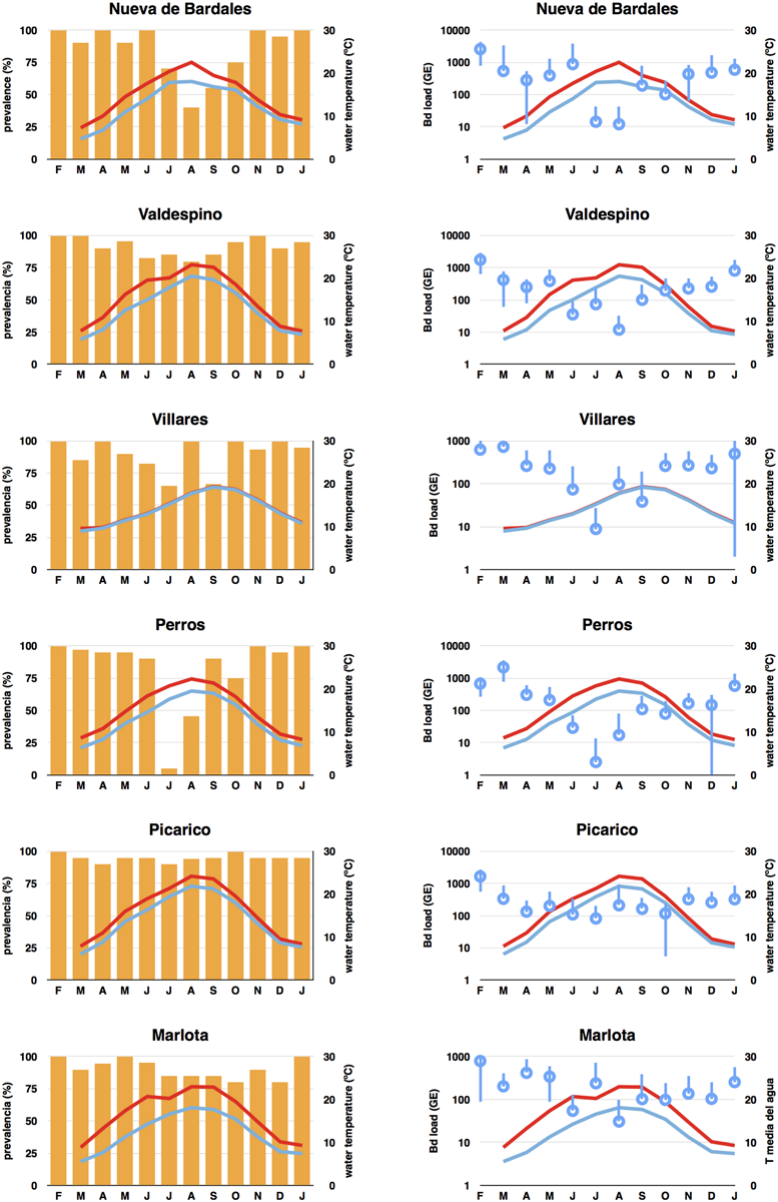
Prevalence and infection load of *Batrachochytrium dendrobatidis*. Prevalence (left) and infection load (right; in logarithm scale, GE, mean +/- SE) for populations of *Alytes obstetricans* tadpoles in six different sites throughout 12 months in each site. Monthly average minimum and maximum water temperature are shown in blue and red respectively.

Seasonal changes in water temperature were very consistent across study sites (see Fig. 1), with lower temperatures from January to March (around 8°C), and higher temperatures in late summer (August and September; around 20°C). The lowest minimum water temperature registered was 1.2°C (March), while the highest recorded maximum water temperature was 25.4°C (September). Maximum differences among sites in average monthly temperatures were around 4°C, ranging from 2.0°C in April to 5.4°C in August; these differences among sites were very similar considering both the monthly minimum and maximum temperatures. Diurnal temperature variations were on average 2.2°C across sites and throughout the study period, with highest recorded figures in June (3.5°C in average) and lowest figures in December and January (1.0°C).

For all sites, the prevalence of *Bd* infection in tadpoles was highly variable, with an average per site ranging from 5 to 100% (Fig. 1). Prevalence was strongly correlated with population averaged *Bd* loads, demonstrating a steep increase from zero to 100 zoospore equivalents, and a nearly saturated prevalence above 200 zoospore equivalents. The relationship between these two variables approached linearity when prevalence was arcsin-transformed and zoospore load was log-transformed. Thus, 54.4% of variation in *Bd* prevalence across sites and throughout months was explained by a model including *Bd* load and site (F_6,59_ = 11.76, p<<0.001); population averaged *Bd* load accounted for 49.6% of variance in *Bd* prevalence (F_1,5_ = 31.47, p = 0.002) while site accounted only for 2.7% of variance (F_5,59_ = 0.70, p = 0.626). For this reason, subsequent data analyses have been restricted to population averaged *Bd* load for the sake of brevity, considering the tight relationship between *Bd* prevalence and load, the very high prevalences measured, and according to the fact that prevalence did not reliably inform about the high variation in *Bd* load with prevalences greater than 75%.

Subsequent addition of minimum and maximum temperatures to models including tadpole abundance, tadpole development and site considerably increased the proportion of variance explained by population averaged *Bd* load, and the strength of evidence of models (Table 1). The model lacking temperature had a strength of evidence many times (>1,000) lower than the models including average minimum and maximum temperatures 2, 5, 10, 15, 22 and 30 days before tadpole sampling. Moreover, models including both temperatures five or two days before sampling occurred had strengths of evidence >200,000 times higher than models not including temperature.

The effect of temperature in explaining population averaged *Bd* load diminished from the short-term (i.e., two days before tadpole sampling) through to mid-term (i.e., 30 days before sampling), shown by the increase in AICc values and the decrease in model weights in Table 1. According to the quotient of model weights, considering average temperatures during the two days prior to tadpole sampling provided a model with a strength of evidence 222 times higher than the model that included average temperatures during the preceding 30 days.

Both minimum and maximum temperatures had a negative influence on population averaged *Bd* load, with the effect of minimum temperature (β = -0.648) being higher than that of maximum temperature (β = -0.047; see standardized regression coefficients and their weighted averages in Table 1). Thus, population averaged *Bd* load decreased across sites and months as temperature increased. Local tadpole abundance and development had a negligible influence on population averaged *Bd* load.

**Fig 2:**
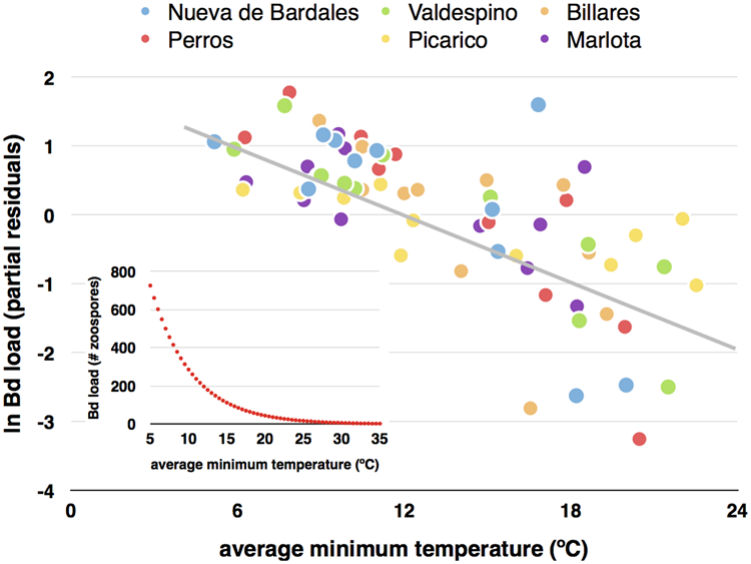
Average minimum water temperature and infection load of *Batrachochytrium dendrobatidis*. Partial residual plot illustrating the influence of average minimum water temperature two days before tadpole sampling, on *Batrachochytrium dendrobatidis* infection load (in logarithm) of *Alytes obstetricans* tadpoles from six different sites. Sample size is 11 months for each site. The residual plot shows the relationship between minimum temperature and *Bd* load given that the other two independent variables are also in the model (see with temperature-2 days in Table 1), therefore, partialling out their effects. The inner panel shows the modeled relationship between *Bd* load (average number of zoospores per tadpole) and average minimum temperature two days before sampling.

The negative influence of average minimum temperature on population averaged *Bd* load steadily decreased from very short time spans of two days to longer periods of 30 days (see Fig. 2 for its influence in the time span of two days before tadpole sampling). The converse pattern was observed for the average maximum temperature, whose importance was higher averaging data for 22 and 30 days prior to tadpole sampling (see also β coefficients in Table 1).

The most parsimonious model, of all those possible, in explaining population averaged *Bd* load was one that included the average minimum temperature two days before tadpole sampling and the factor “site” (AICc = 173.7; R^2^ = 0.522; F_6,59_ = 10.73, p<<0.001), with a strong negative effect of minimum temperature (β = -0.696; F_1,5_ = 25.46, p<0.001) and “site” explaining a low amount of variance (5.1%; F_5,59_ = 1.60, p = 0.174). A similarly parsimonious model (AICc = 173.9; R^2^ = 0.520; F_6,59_ = 10.65, p<<0.001) included minimum temperature five days before tadpole sampling (β = -0.694) and site as predictors (5.1% of variance). The first model, including the average maximum temperature during 30 days before tadpole sampling, had a considerably lower strength of evidence (AICc = 184.2; R^2^ = 0.439; F_6,59_ = 7.69, p<<0.001), with temperature having a strong negative effect (β = -0.631; F_1,5_ = 22.39, p<0.001) and “site” explaining 4.9% of variance (F_5,59_ = 1.19, p = 0.326).

The proportion of variability in population averaged *Bd* load not accounted by the six a priori models in Table 1 including temperature (i.e., model residuals) was explained to a very low extent by sampling date (using a cubic polynomial of month: 0.8–2.2%; p>0.71 in the six models).

In summary, temperature had an important role in determining the intensity of infections by *Bd*. Its effect was considerably more important when the average minimum temperature 2–5 days before tadpole sampling was taken into account, and the influence of average minimum temperature on population averaged *Bd* load was considerably higher than that recorded for the average maximum temperature. Variability in population averaged *Bd* load that could be attributed to different localities was low, accounting for only 5% of the total variability. The influence of tadpole abundance and tadpole development on population averaged *Bd* loads was nearly negligible and the proportion of variability in population averaged *Bd* loads that was accounted by sampling date was very low.

## Discussion

Previous studies of *Bd*-infected *Alytes muletensis* tadpoles on the island of Mallorca showed that the survival of infected populations was site specific, and that this owed to the role of temperature in regulating the host/pathogen dynamic [20]. In our current study, we demonstrate across a replicated set of mainland *A*. *obstetricans* breeding sites that temperature exerts a similarly profound effect. This leads to pronounced seasonal cyclicity in *Bd* loads, with the highest burdens of infection seen during the colder months. In other ecosystems various factors such as habitat type, density of the host, life history traits and virulence of the infecting strain of *Bd* have also been argued as possible causes of this variation in host susceptibility [14, 47, 48]. Local weather conditions and their seasonal variation are known to have a large influence on pathogen-host dynamics [22, 39, 42, 48]. As seasonality is typically stronger in temperate climates, where numerous and serious cases of amphibian mass mortalities due to chytridiomycosis have been recorded, some authors have suggested that temperate zones are exposed to a higher risk of outbreaks of chytridiomycosis [49]. This argument has some validity as temparate populations of *Rana muscosa* and *R*. *sierra*, which like *A*. *obstetricans*, are highly vulnerable to chytridiomycosis, show high mortality at high-altitudes [17, 32]. However, in contrast to our findings, these studies have shown no effect of seasonality on the intensity of *Bd* infections. This likely owes to the fact that in these montane areas of the USA researchers cannot sample amphibians in the winter months due to the presence of the snow cover; our sample sites fail to freeze in winter and we are able to sample throughout the year.

We witnessed increasing infection loads while temperature decreased, *i*.*e*. peaks of infection in the winter. Many other authors have obtained similar results in the laboratory [38, 50, 51, 52] and also in the field, however studies have most often been undertaken in tropical areas, or have not covered the entire seasonal variation in temperature [22, 32, 33, 36, 53, 54]. Our study corroborates these results in a temperate climate, by monitoring the infection on tadpoles monthly during a year in the field. The higher importance of minimum over maximum water temperature is easily understandable considering that maximum temperatures allow for the availability of lower temperatures throughout the day that are more favorable for the chytrid fungus.

We found that the effect of average minimum temperature was higher than that recorded for the maximum temperature during the same period of time. Although the range of averaged maximum temperatures recorded during summer months (15.3–23.6°C) was within the optimal range of *Bd* growth (17–25°C)[28], we observed the lowest infection loads across this period. We have previously used mathematical models and analysis of the temperature-dependent expression of *Bd*-infected adult *Silurana tropicalis* immune-related genes to show that temperature, zoospore growth rates and immune-related clearance all interact to determine *Bd* loads [55]. While our current study is in focused on larval, rather than adult, stages, it is likely that a similar interaction between host and pathogen life-history variables are interacting with temperature to determine the intensity of infections. During spring and autumn we saw similar ranges of averaged maximum temperatures (9.8–20.7°C and 8.9–19.5°C, respectively) and also similar infection intensity between these two seasons. While the range of temperatures during winter (7.3–12.6°C) was outside of the optimal range for *Bd*, we saw the highest infection loads precisely in this season. This suggests a failure in the ability of the tadpoles to clear infection, rather than the effect of the pathogens growth alone. The increased susceptibility that we see is likely related to the poorer functioning of the immune system at temperatures below 10°C, a fact that has been strongly supported in a study of temperature-dependent immune inhibition in *S*. *tropicalis* [55]. However, far less is known about the temperature-dependency of immune function in larval amphibians and here we can only speculate that our findings may owe to temperature-dependent immune-inhibition, that may impact the adaptive and innate arms of the immune system including tadpole ability to synthesise antimicrobial peptides. That we see the greatest impact of temperature just before sampling episodes shows that these temperature-related impacts on susceptibility are dynamic. However, further work is required to disentangle the causal relationships between rates of *Bd* growth and tadpole immune-responses.

Partialling out the effects of the other variables, average minimum water temperature two days before sampling shows a strong negative relationship with the logarithm of average population *Bd* load (see the inner panel in Fig. 2). The lower 95% confidence interval of the modeled relationship makes the infection equal to zero zoospores when the average minimum temperature is 25°C (a very similar relationship and temperature cut-off point is obtained analyzing the time span of 5-days before tadpole sampling). Nevertheless, the minimum water temperature predicted for completely eliminating *Bd* infection at the population level was 35°C. Therefore, if minimum water temperature is higher than 25°C during two-five days before, there is a very high probability that most, but not all, tadpoles lose the infection. This result matches those previously found in *A*. *obstetricans* in Switzerland [56], and confirm the valid cut-off point of ca. 30°C to completely eliminate *Bd* infection in the wild considering that above this temperature *Bd* in culture begins to die [24, 54].

We found that tadpole abundance and tadpole development exert a negligible effect on *Bd* infection and, in agreement with our findings, detailed studies of *R*. *muscosa* tadpoles [57] also failed to find a significant relationship between *Bd* presence and larval stage. On the other hand, some laboratory and field studies [58] indicate a clear influence of the density of infected individuals in the rates of *Bd* transmission. Despite the fact that some of these studies considered *Bd* prevalence while others *Bd* load, these contrasting results could be related to differences among species in their intrinsic susceptibility to infection by *Bd* and requires further study.

Determining the relationship between environmental variables across local scales and their relationship to pathogen growth, disease infection levels, and ultimately episodes of mass mortality, will allow us to improve our ability to model and predict the impacts of this infection across host populations. This will be useful, for example, in refining survey strategies by selecting the periods with the highest burdens of infection and, therefore, reducing sample size required without losing statistical power. Additionally, and more importantly, this knowledge will improve our ability to make evidence based management decisions to undertake disease mitigation attempts during the periods with the lowest burdens of infection. Ultimately, this will enhance the decision making process with regard to conservation measures that could enhance the survival of endangered or threatened species.
